# Perception of Quiet Students in Emergency Medicine: An Exploration of Narratives in the Standardized Letter of Evaluation

**DOI:** 10.5811/westjem.57756

**Published:** 2023-07-12

**Authors:** John K. Quinn, Jillian Mongelluzzo, Alyssa Nip, Joseph Graterol, Esther H. Chen

**Affiliations:** University of California, San Francisco, Department of Emergency Medicine, San Francisco, California

## Abstract

**Introduction:**

The Standardized Letter of Evaluation (SLOE) is designed to assist emergency medicine (EM) residency programs in differentiating applicants and in selecting those to interview. The SLOE narrative component summarizes the student’s clinical skills as well as their non-cognitive attributes. The purpose of this qualitative investigation was to explore how students described in the SLOE as quiet are perceived by faculty and to better understand how this may impact their residency candidacy.

**Methods:**

This retrospective cohort study included all SLOEs submitted to one EM residency program during one application cycle. We analyzed sentences in the SLOE narrative describing students as “quiet,” “shy,” and/or “reserved.” Using grounded theory, thematic content analysis with a constructivist approach, we identified five mutually exclusive themes that best characterized the usage of these target words.

**Results:**

We identified five themes: 1) quiet traits portrayed as implied-negative attributes (62.4%); 2) quiet students portrayed as overshadowed by more extraverted peers (10.3%); 3) quiet students portrayed as unfit for fast-paced clinical settings (3.4%); 4) “quiet” portrayed as a positive attribute (10.3%); and 5) “quiet” comments deemed difficult to assess due to lack of context (15.6%).

**Conclusion:**

We found that quiet personality traits were often portrayed as negative attributes. Further, comments often lacked clinical context, leaving them vulnerable to misunderstanding or bias. More research is needed to determine how quiet students perform compared to their non-quiet peers and to determine what changes to instructional practices may support the quiet student and help create a more inclusive learning environment.

## INTRODUCTION

The emergency medicine (EM) Standardized Letter of Evaluation (SLOE) is a high-stakes assessment designed to assist residency programs in differentiating applicants and is considered important in the decision to interview.^1^,^2^ The narrative component summarizes the student’s knowledge, clinical skills, and non-cognitive attributes shown to be predictors of performance.^3^–^5^ However, the narrative may be difficult to interpret due to the use of overly general language and hidden code, both common in written assessment.^6^–^9^ Further, comments about personality often lack clinical context, which reduces their usefulness and makes them vulnerable to misinterpretation or bias.^6^–^8^

We became interested in SLOE narratives referencing quiet students during applicant review when we observed less enthusiasm for students described as quiet, even for those with strong objective application data. While non-cognitive attributes are important components of holistic assessment, personality traits should not necessarily hinder a strong application.^3^–^5^ No studies show that quiet individuals are unsuited for EM or are less successful in EM careers. However, in an internal medicine setting, “quiet” was interpreted by attendings as a “red flag” in clerkship written evaluations, 9,10 and students described as quiet in their SLOE scored lower on both global assessment and anticipated rank list.^11^ We found no other research examining how quiet individuals perform or how they were perceived in EM. The purpose of this qualitative investigation was to explore how quiet students are described in the SLOE narrative and how this language may impact candidacy.

## METHODS

### Study Design and Population

We conducted a subgroup analysis of a retrospective cohort study of all core EM rotation SLOEs submitted through the Electronic Residency Application Service (ERAS) to one EM residency program during the 2016–2017 application cycle. We excluded SLOEs from non-Liaison Committee on Medical Education accredited schools and applicants who graduated from medical school before or during the application cycle. The study was approved by the institutional review board and the Association of American Medical Colleges.

### Study Protocol and Data Analysis

Author JM downloaded SLOEs from ERAS into REDCap (Research Electronic Data Capture tools hosted at UC San Francisco). and de-identified them prior to analysis. Analysis was performed by JKQ, EHC, and JM, all with training in medical education research methodology and education leadership experience (chief resident, associate residency director, and assistant residency director). JKQ and EHC brainstormed words typically used to describe quiet individuals and chose the target-descriptors quiet, shy, and reserved (collectively termed “quiet”) because passive, introverted, and timid were uncommon (3, 2, and 1, respectively) and always co-occurred with target-descriptors. We analyzed only the sentence containing the target-descriptors without exploring the entire narrative. We analyzed data using grounded theory thematic content analysis with a constructivist approach.^12^ There was no pre-existing theory about the data that we aimed to prove or disprove; instead, the goal was to explore SLOE narrative-comments and construct meaning from them to provide perspective on how quiet students are perceived.

Without a preset idea of how data would be sorted, JKQ and EHC independently began the initial coding by reading each comment and considering how it was used to describe the student. As usage patterns emerged they were coded as like-comments. JM read a subset of the data. To establish that the dataset was sufficient for the purpose of the investigation, we coded the first half of the dataset and then determined that no new patterns emerged in the second half. We progressed to explaining our coding schemes, comparing them, and looking for similarities and differences. Through an iterative process of constant comparison we combined, deleted, and refined codes, merging them into overarching themes. We used a spreadsheet to visually organize codes and final themes.

## RESULTS

We reviewed 1,582 SLOEs from 696 applicants. Of these, 117 SLOEs referenced quiet applicants and were analyzed. The adjective “quiet” occurred in 102 SLOEs. “Reserved” occurred in 28 SLOEs and co-occurred 14 times with “quiet.” “Shy” occurred in 11 SLOEs and co-occurred five times with “quiet.”

Initial coding revealed usage related to interpersonal skills, initiative, disposition, patient interactions, leadership, medical knowledge, response to feedback, work habits, and fitness for EM. Further analysis revealed that many target sentences did not fit into these categories, lacked clinical context, and were difficult to interpret. We eventually reached a consensus on a framework of five mutually exclusive overarching themes that included all comments, best represented the scope of usage patterns, and would be most meaningful in addressing our study purpose (Table 1).

Theme 1 comments, 62.4% describe quiet traits as implied-negative attributes. Comments are labeled “implied” because quiet is not explicitly called negative but is typically coupled with a contrasting positive trait that appears to be an effort to mitigate the negativity of the quiet comment (eg, “Quiet but hardworking”). The structure of the sentence makes it clear that quiet is negative, but it is not evident in what way or to what degree it is negative. A smaller number of comments linked the quiet trait with another seemingly negative attribute (eg, “Quiet and timid at times”). The implied negativity of these comments coupled with the lack of context may adversely affect the applicant’s candidacy.

Theme 2 comments (10.3%) describe quiet students as being overshadowed by more extraverted peers and thus more difficult to assess. These comments also did not explain how performance was impacted by the quiet trait, only that the student was not able to demonstrate value as a candidate or perform at the level of their peers, which presumably hinders applicant candidacy.

Theme 3 comments 3.4%) question the fitness of quiet students for fast-paced clinical settings. However, these comments did not detail how, or to what degree, the student’s quietness specifically affected performance, making them vulnerable to misinterpretation. These comments would likely also hinder candidacy, as the ability to perform well in all clinical settings is presumably seen as necessary in a successful EM resident.

Theme 4 comments (10.3%) “quiet” is portrayed as a positive attribute and tends to describe leadership style, patient interactions, or ability to perform under pressure, rather than describing student personality. This additional context may have contributed to the overall perception of “quiet” as a positive attribute. Theme 5 comments (15.6%) were considered equivocal in that the investigators either did not agree on the positivity or negativity of their interpretation, or the comments lacked sufficient context to interpret the intended meaning (eg, “Student was initially quiet”).

## DISCUSSION

We found that quiet traits were usually portrayed as negative attributes and, therefore, had the potential to adversely affect the candidacy of a considerable number of applicants. The analysis also revealed that across themes the quiet trait was rarely described in terms of clinical competency. This is concerning because a negative comment that lacks context requires the reader to rely on inferences or assumptions that may result in unfairly judging the applicant. Providing examples that describe observed behavior and clinical skill, rather than referencing personality, will improve the quality and fairness of the assessment.^6^,^7^

Our findings that quiet students are described as being overshadowed by more extraverted peers, more difficult to assess, and less fit for fast-paced clinical settings suggest the possibility that current instructional practices favor more outgoing students. In a clinical setting where being assertive is viewed favorably, quiet students may be judged unfairly as being less knowledgeable or prepared.^3^,^13^ Changes to instructional practices that may better serve quiet students include the following: providing additional student observations^6^; using standardized assessment-tools^14^,^15^; expanding assessment criteria to include strengths of the introvert^13^; providing faculty development to improve quality of written assessment^7^; using group-written SLOEs that may reduce bias^1^,^2^; and providing student mentorship.^3^

## LIMITATIONS

This study was limited to SLOEs from applicants to a single institution during one application cycle. We analyzed only the sentence containing the target-descriptors; reading the entire narrative may have provided additional context. Target-descriptors may be defined differently by different evaluators and readers and may or may not be used interchangeably. Further, readers may interpret the positivity or negativity of the usage differently than the investigators. The target-descriptors may not reflect student personality but rather how they were perceived by their evaluator in the clinical setting. Applicants did not receive a personality inventory nor did they self-report their personality type. We did not identify the gender of applicant or the SLOE writer, which prevented us from determining whether our findings were affected by gender. Nor did we identify the position or experience of the writer, or whether individual or group process was used. We did not attempt to associate quiet vs non-quiet status with an invitation to interview.

## CONCLUSION

We found that quiet personality traits were often portrayed as negative attributes in the Standardized Letter of Evaluation. Additionally, clinical context was rarely provided, leaving comments open to variable interpretation and possible misunderstanding of student competency. These findings add to our understanding about quiet students in EM, but more research is needed to determine how quiet-labeled students perform compared to their non-quiet peers and to determine what changes to instructional practices may support the quiet student and help create amore inclusive learning environment where all students can thrive.

## Figures and Tables

**Table 1:** Thematic analysis of 117 sentences containing the words “quiet”, “shy” or “reserved”.

Theme	Subthemes	Examples
Theme 1) Implied negative (n = 73)	1A) Quiet nature is mitigated by associating with a positive interpersonal skill.	“Quiet but was always able to communicate effectively.” “Somewhat reserved but can be assertive when necessary.”
	1B) Quiet nature is mitigated by associating with a positive attribute unrelated to quiet personality.	“Quiet but hardworking.” “Can be reserved at times but is incredibly intelligent.”
Theme 2) Quiet students may be overshadowed by others (n = 12)	2A) Quiet students overshadowed by more extraverted students.	“Quiet demeanor and presence of flashier students prevented a higher ranking.” “Overshadowed, quieter than peers, disappeared into background most of the month.”
	2B) Quiet students’ clinical skills difficult to assess due to their quiet personality.	“Truncated presentations and quiet demeanor make it difficult to evaluate true potential.” “So quiet I could not judge level of engagement.”
Theme 3) Quiet students may be less suited for certain clinical settings (n = 4)	3A) Quiet students perceived as too passive, slow, or unassertive for a busy clinical setting.	“Quiet, passive nature may not be suited for high paced inner-city ED.” “Quiet and unassuming personality, some noted this to be a concern, particularly in a busy county ED, others didn’t.”
	3B) Quiet students perceived as less adaptable to the demands of a busy clinical setting.	“Calm, quiet, reserved demeanor-some staff question adaptability to chaotic ED.”
Theme 4) Positive trait (n = 12)		“Soothing demeanor and quiet confidence will suit quite well throughout their career.” “Quiet demeanor, kind bedside manner which is an asset with patients.”
Theme 5) Equivocal (n = 16)		“A little quiet, we do not think this will hinder ability to be a very capable EM resident.” “Quiet”

*ED*, emergency department; *EM*, emergency medicine.

## References

[1] Love JN, Doty CI, Smith JL (2020). The emergency medicine group Standardized Letter of Evaluation as a workplace-based assessment: The validity Is in the detail. West J Emerg Med.

[2] Negaard M, Assimacopoulos E, Harland K (2018). Emergency medicine residency selection criteria: an update and comparison. AEM Educ Train.

[3] Khan MA, Malviya M, English K (2021). Medical student personality traits and clinical grades in the internal medicine clerkship. Med Sci Educ.

[4] Pines JM, Alfaraj S, Batra S (2018). Factors Important to top clinical performance in emergency medicine residency: results of an ideation survey and Delphi panel. AEM Educ Train.

[5] Sobowale K, Ham SA, Curlin FA (2018). Personality traits are associated with academic achievement in medical school: a nationally representative study. Acad Psychiatry.

[6] Jackson JL, Kay C, Jackson WC (2015). The quality of written feedback by attendings of internal medicine residents. J Gen Intern Med.

[7] Ledford R, Burger A, LaRochelle J (2020). Exploring perspectives from internal medicine clerkship directors in the USA on effective narrative evaluation: results from the CDIM national survey. Med Sci Educ.

[8] Lye PS, Biernat KA, Bragg DS (2001). A pleasure to work with–an analysis of written comments on student evaluations. Ambul Pediatr.

[9] Ginsburg S, Kogan JR, Gingerich A (2020). Taken out of context: hazards in the interpretation of written assessment comments. Acad Med.

[10] Ginsburg S, McIlroy J, Oulanova O (2010). Toward authentic clinical evaluation: pitfalls in the pursuit of competency. Acad Med.

[11] Quinn JK, Mongelluzzo J, Addo N (2023). The Standardized Letter of Evaluation: how we perceive the quiet student. West J Emerg Med.

[12] Coates WC, Jordan J, Clarke SO (2021). A practical guide for conducting qualitative research in medical education: Part 2-Coding and thematic analysis. AEM Educ Train.

[13] Davidson B, Gillies RA, Pelletier AL (2015). Introversion and medical student education: challenges for both students and educators. Teach Learn Med.

[14] Davis KR, Banken JA (2005). Personality type and clinical evaluations in an obstetrics/gynecology medical student clerkship. Am J Obstet Gynecol.

[15] Schell RM, Dilorenzo AN, Li HF (2012). Anesthesiology resident personality type correlates with faculty assessment of resident performance. J Clin Anesth.

